# GPR15LG regulates psoriasis-like inflammation by down-regulating inflammatory factors on keratinocytes

**DOI:** 10.1042/BSR20231347

**Published:** 2024-05-31

**Authors:** Caifeng Chen, Renhui Cai, Jun Zhou, Danqun Zhang, Li Chen

**Affiliations:** Department of Dermatology, Fujian Provincial Hospital, Clinical Medical College of Fujian Medical University, Fujian Fuzhou, China

**Keywords:** GPR15LG, inflammation, keratinocytes, psoriasis

## Abstract

Psoriasis is a common chronic inflammatory skin disease characterized by aberrant proliferation of keratinocytes and infiltration of immune cells. We previously found that *GPR15LG* protein is highly expressed in psoriasis lesional skin and it positively regulates psoriatic keratinocyte proliferation. Our data also showed that GPR15LG could regulate the activity of NF-κB pathway, which is associated with psoriatic inflammation. In the present study, we demonstrated that *Gpr15lg* (ortholog of *GPR15LG*) knockdown attenuated the severity of imiquimod (IMQ)-induced psoriasis-like inflammation in mice. Such an effect was achieved by down-regulating the expression of inflammatory cytokines interleukin (IL)-1α, IL-1β, tumor necrosis factor (TNF)-α and S100A7. Consistently, *GPR15LG* knockdown *in vitro* significantly downgraded the expression of inflammatory factors in the cellular model of psoriasis. These results suggested that GPR15LG could be involved in the development of psoriasis by regulating inflammation.

## Introduction

Psoriasis is a chronic immune-mediated skin disorder, affecting 2–4% of the world’s population [1,2]. Psoriasis manifests as scaly erythematous plaques [1,2]. Patients with psoriasis are at an increased risk of developing several comorbidities [3,4]. They experience a reduction in the life quality with substantial economic burden and psychological burden [5]. It is characterized by epidermal hyperplasia and intense inflammation [6]. The exact pathogenesis of psoriasis is still not fully understood and available treatments are not absolutely effective. Therefore, more research is need to further elucidate the pathogenesis of psoriasis.

GPR15LG is a human antimicrobial peptide expressed in epithelial tissues [7,8]. GPR15LG has been shown to modulate a variety of cellular functions and several functions of GPR15LG have been found in the context of psoriasis. However, to our knowledge, the capability of GPR15LG on regulating psoriasis-like skin inflammation remains largely unknown.

In the present study, the IMQ-induced psoriasis-like mouse model and M5-induced cellular model of psoriasis were employed to investigate the role of GPR15LG in psoriatic inflammation *in vivo* and *in vitro*.

## Materials and methods

### Cell line

HaCaT and the mouse muscle cell line C2C12 were cultured in DMEM supplemented with 1% penicillin/streptomycin and 10% FBS. Cells were kept in a humidified incubator at 37°C with 5% CO_2_.

### Induction of psoriatic model *in vitro*

HaCaT cells were stimulated with 10 ng/ml recombinant IL-1α, TNF-α, OSM, IL-17A and IL-22 (Peprotech, USA) alone or in combination (named M5 cytokines cocktail) to induce psoriatic inflammation.

### Lentivirus transduction

The oligonucleotides of shRNAs were listed in Table 1. HaCaT cells were incubated with virus suspension for 48 h and puromycin was used to screen the stable infected cells for 14 days.

**Table 1:** The oligonucleotides of shRNAs

Name	Sequences
siRNA-1	Forward:5′-CAUCUUCUCCACAGAAGGGAATT-3′
	Reverse: 5′-UUCCCUUCUGUGGAGAAGAUGTT-3′
siRNA-2	Forward 5′-GACAUCAUGUGAGGCUCUGUATT-3′
	Reverse 5′-UACAGAGCCUCACAUGAUGUCTT-3′
siRNA-3	Forward: 5′- GCCAUCAACUUUCAGAGCUAUTT-3′
	Reverse: 5′- AUAGCUCUGAAAGUUGAUGGCTT-3′
si-NC	Forward:5′-UUCUCCGAACGUGUCACGUTT-3′
	Reverse: 5′-ACGUGACACGUUCGGAGAATT-3′

### RNA extraction and qRT-PCR

Total RNA was extracted from cells or tissues using TRIzol (CWBIO) following the manufacturers’ instructions. cDNA was synthesized with the kit (R223-01, Vazyme). qRT-PCR was done using SYBR qPCR Master Mix (Vazyme). The primer sequences used in the experiment were shown in Table 2.

**Table 2:** Primer sequences for qRT-PCR

Gene name	Primer sequence (5′−3′)
GAPDH	Forward 5′-TGTTGCCATCAATGACCCCTT-3′
	Reverse 5′-CTCCACGACGTACTCAGCG-3′
TNF-α	Forward 5′-CGAGTGACAAGCCTGTAGCC-3′
	Reverse 5′-TGAAGAGGACCTGGGAGTAGAT-3′
IL-1α	Forward 5′-TTGTATGTGACTGCCCAAGAT-3′
	Reverse 5′-TCCCAGAAGAAGAGGAGGTT-3′
IL-1β	Forward 5′-GCACGATGCACCTGTACGAT-3′
	Reverse 5′-TGGAGAACACCACTTGTTGC-3′
S100A7	Forward 5′-CCAACTTCCTTAGTGCCTGTG-3′
	Reverse 5′-GCTCTGCTTGTGGTAGTCTGTG-3′
Gpr15lg	Forward 5′-GAGACTTCTAGCCCTTTCCG-3′
	Reverse 5′-TGGTTTCCTTTCCAGGTTGT-3′
Mouse GAPDH	Forward 5′-TCAACGGCACAGTCAAGG-3′
	Reverse 5′-TGAGCCCTTCCACGATG-3′
Mouse TNF-α	Forward 5′-CAGGCGGTGCCTATGTCTC-3′
	Reverse 5′-CGATCACCCCGAAGTTCAGTAG-3′
Mouse IL-1α	Forward 5′-CCCGTGTTGCTGAAGGAGTTG-3′
	Reverse 5′-CTGTCATAGAGGGCAGTCCC-3′
β-Actin	Forward 5′-TGGCACCCAGCACAATGAA-3′
	Reverse 5′-CTAAGTCATAGTCCGCCTAGAAGCA-3′
GPR15LG	Forward 5′-GCTTCTCTGCTTCTCCATCTTCT -3′
	Reverse 5′-TTCAGGTTTGTTGAGTTGGG-3′

### Mice

Female BALB/c mice (8 weeks of age) were acclimatized for 1 week with free access to food and water. The study was approved by the Ethics Committee of Fujian Provincial Hospital (approval number: K2019-03-056) and all experimental procedures were performed in accordance with the Guide for the Care and Use of Laboratory Animals of the National Institutes of Health.

### Animal experiments

Mice were randomly divided into the following four groups: Control group (Ctr, *n*=6), IMQ group (IMQ, *n*=6), IMQ + sh-NC group (IMQ+sh-NC, *n*=6), IMQ + shRNA-1 group (IMQ+sh-1, *n*=6). Mice in IMQ + sh-NC group and mice in IMQ + sh-1 group were injected intradermally with lentivirus particles (1.0 × 10^9^ TU, 50 µl) encoding negative control shRNA or *Gpr15lg* shRNA. The oligonucleotides of shRNAs were listed in Table 3. Three days after adenovirus particles treatment, groups, except the control group, were topically administered with 62.5 mg of 5% IMQ cream on the shaved back for 7 days. After treatment with IMQ, the mice were killed and we collected the dorsal skin samples. Half samples was fixed in formalin prepared for histological evaluation and immunohistochemistry and other tissues were frozen in liquid nitrogen for further detection.

**Table 3:** The oligonucleotides of shRNAs

Name	Sequences
siRNA-1	Forward: 5′-CAGAAACAAGCTACCAGTCAAGTCATT-3′
	Reverse: 5′-UGACUUGACUGGUAGCUUGUUUCUGTT-3′
siRNA-2	Forward: 5′-TCTGCAGAAACAAGCTACCAGTCAATT-3′
	Reverse: 5′-UUGACUGGUAGCUUGUUUCUGCAGATT-3′
siRNA-3	Forward: 5′-UAGAUCCAAGCUGACAACCUGGAAATT-3′
	Reverse: 5′-UUUCCAGGUUGUCAGCUUGGAUCUATT-3′
si-NC	Forward:5′-UUCUCCGAACGUGUCACGUTT-3′
	Reverse: 5′-ACGUGACACGUUCGGAGAATT-3′

### Evaluation severity of skin inflammation

Psoriasis Area Severity Index (PASI) was used to score the mice skin inflammation severity. Scales, erythema and thickness were scored independently from 0 to 4. The cumulative score was obtained from the sum of the above three parameters.

### Histological evaluation and Immunohistochemistry

The skin samples from each group were fixed in formalin for 24 h and 5 μm-thickness paraffin sections were stained with H&E. The cell layers of the epidermis and inflammatory cells were counted under high-power fields. Immunohistochemistry (IHC) was performed according to standard methods. For immunohistochemical staining, sections were incubated with specific primary antibodies against IL-1α (YT232), IL-1β (YT2322) and S100A7 (YT6273).

### Statistical analysis

All data were presented as mean ± SEM from at least three independent experiments. Statistical analysis was carried out with GraphPad Prism 5.0. Student’s *t* test was used to compare differences. *P*<0.05 was considered as statistically significant.

## Results

### *Gpr15lg* knockdown ameliorates IMQ-induced psoriatic inflammation in mice

**Figure 1:**
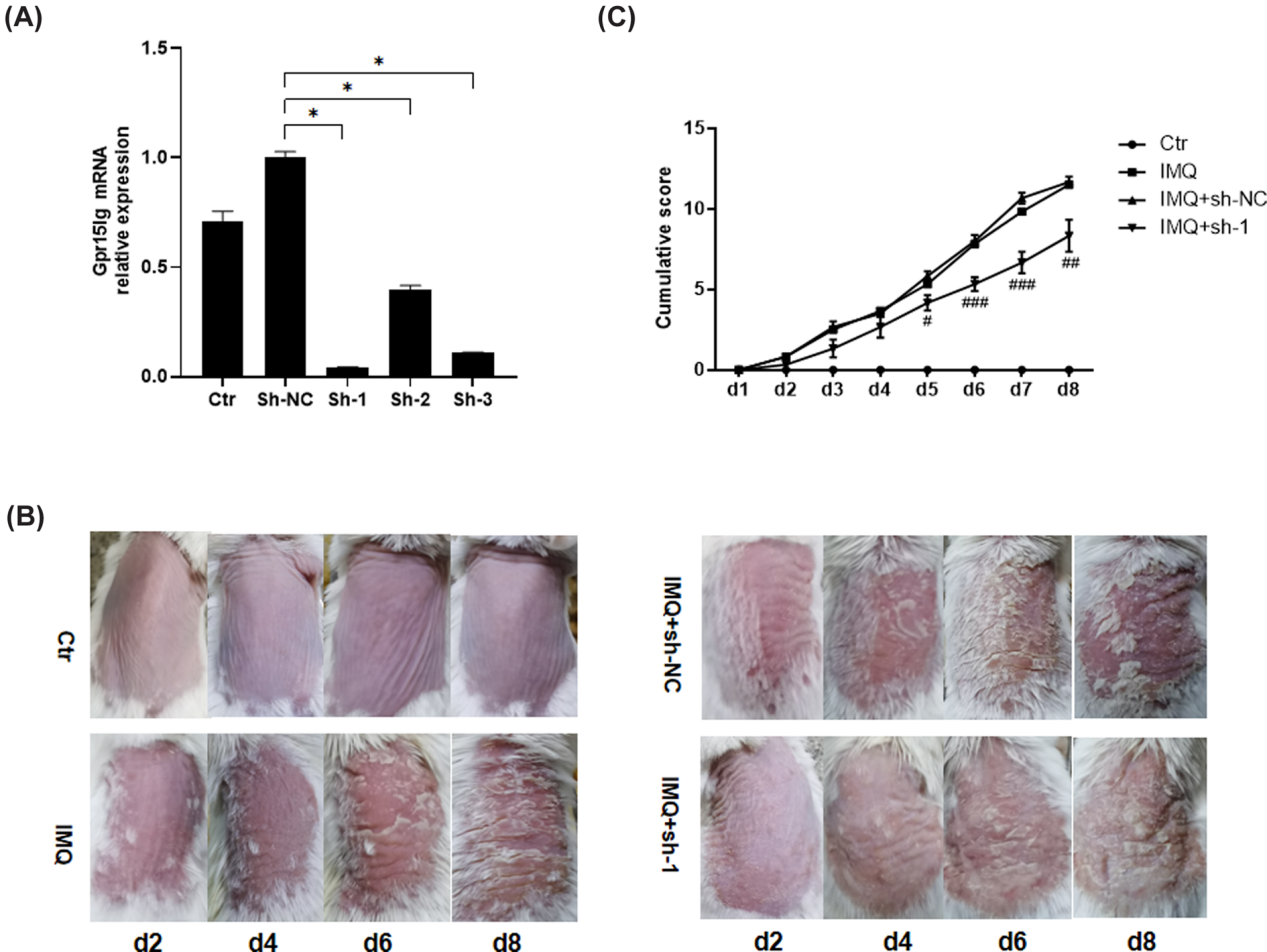
The effect of *Gpr15lg* knockdown on IMQ-induced psoriasis-like lesions (**A**) *Gpr15lg* mRNA was measured in C2C12 transfected with the *Gpr15lg* shRNAs (sh-1 or sh-2 or sh-3) or shRNA-NC (sh-NC). (**B**) Representative clinical pictures of mice back skins on days 2, 4, 6 and 8 of each group after treatment with IMQ. (**C**) Cumulative score was scored everyday based on the PASI (*n*=6). *P<0.05 vs. sh-NC group. #*P*<0.05, ##*P*<0.01, ###*P*<0.001 vs. IMQ+sh-NC group.

To explore the function of GPR15LG in IMQ-induced psoriatic inflammation, we locally knocked down *Gpr15lg* (the mouse ortholog of *GPR15LG*) expression in mouse back skin by injecting the adenoviral particles expressing shRNAs. The knockdown efficiencies of shRNAs were first investigated in C2C12 cells. As shown in Figure 1A, shRNA-1 showed the best knockdown efficiency among three shRNAs and it was chosen for the following animal experiment. IMQ treatment induced typical psoriasis-like lesions (Figure 1B). However, compared with IMQ+sh-NC group, *Gpr15lg* knockdown ameliorated the IMQ-induced mice skin condition (Figure 1B). In addition, we scored the severity of lesions on days 2, 4, 6 and 8 based on PASI. The PASI score of the IMQ group was significantly higher than that of control mice and the mice in IMQ+sh-1 group had lower score than IMQ+sh-NC group (Figure 1C). These results suggest that *Gpr15lg* knockdown could significantly attenuate the IMQ-induced psoriasis-like inflammation in mice.

### *Gpr15lg* knockdown alleviates the morphologies of psoriatic skin inflammation in histopathological analysis

**Figure 2:**
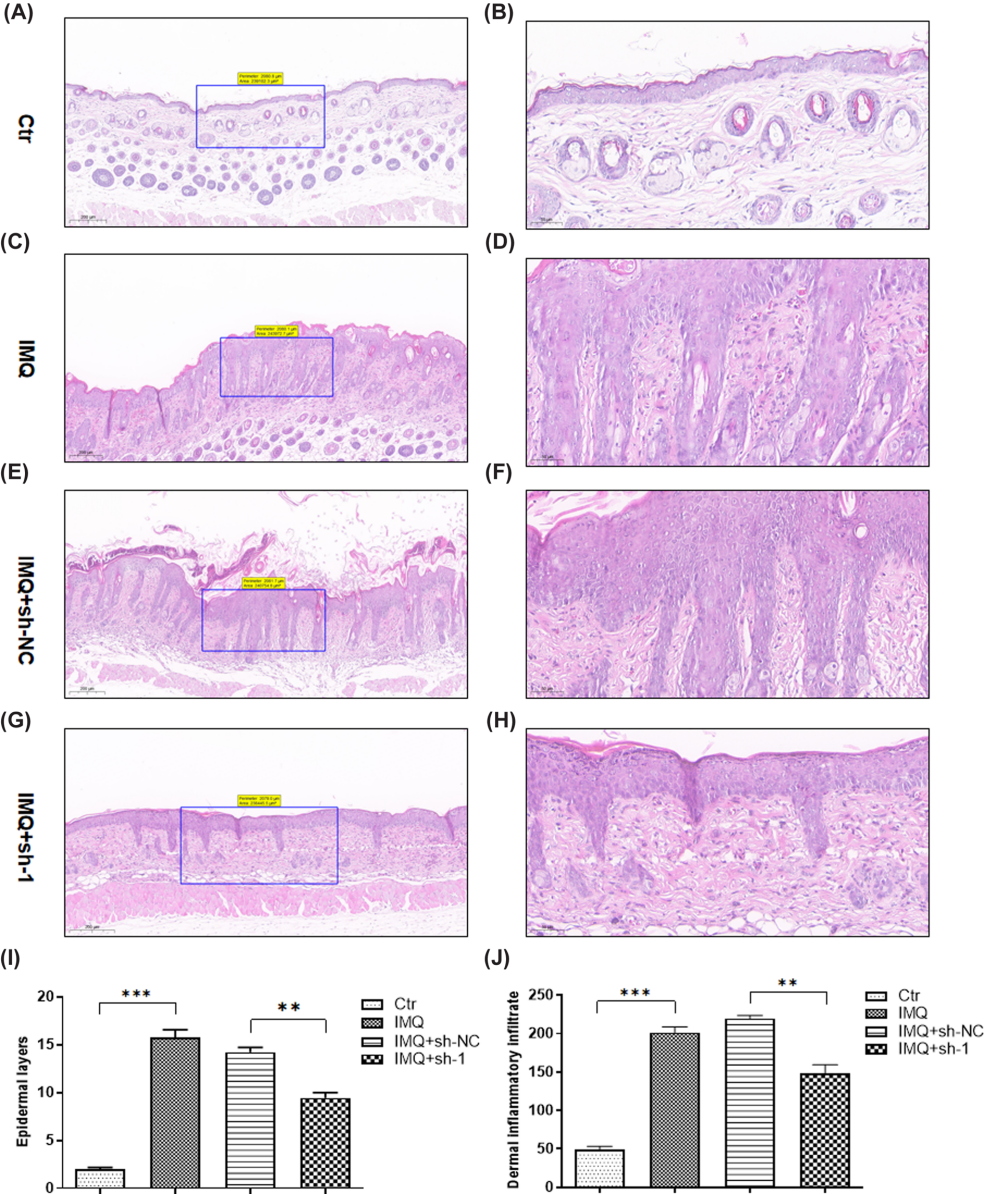
*Gpr15lg* knockdown improves IMQ-induced psoriasis-like skin inflammation histologically (**A**) H&E staning of mice skin tissues in the control group (bar length = 200 μm). (**B**) Magnifcation of the black box in image (A) (bar length = 50 μm). (**C**) H&E staning of mice skin tissues in the IMQ group (bar length = 200 μm). (**D**) Magnifcation of the black box in image (**C**) (bar length = 50 μm). (**E**) H&E staning of mice skin tissues in the IMQ+sh-NC group (bar length = 200 μm). (**F**) Magnifcation of the black box in image (**E**) (bar length = 50 μm). (**G**) H&E staning of mice skin tissues in the IMQ+sh-1 group (bar length = 200 μm). (**H**) Magnifcation of the black box in image (**G**) (bar length = 50 μm). (**I**) The numbers of epidermal layers and (**J**) dermal inflammatory infiltrates on H&E staining were counted under ×400 high-power fields; **P<0.01, ***P<0.001.

We carried out HE staining to further analyzed the lesions. Histopathological analysis showed the mice treated with IMQ had epidermal hyperplasia and inflammatory cells accumulation (Figure 2A–H). The number of cell layers and the number of inflammatory cells were further calculated. The data demonstrated that sh-1 treatment resulted in significant alleviation in the above two indexes (Figure 2I,J). These data showed that *Gpr15lg* knockdown alleviated the histopathological morphologies of psoriasis-like inflammation.

### *Gpr15lg* knockdown reduced levels of inflammatory cytokines in mice psoriatic lesion

**Figure 3:**
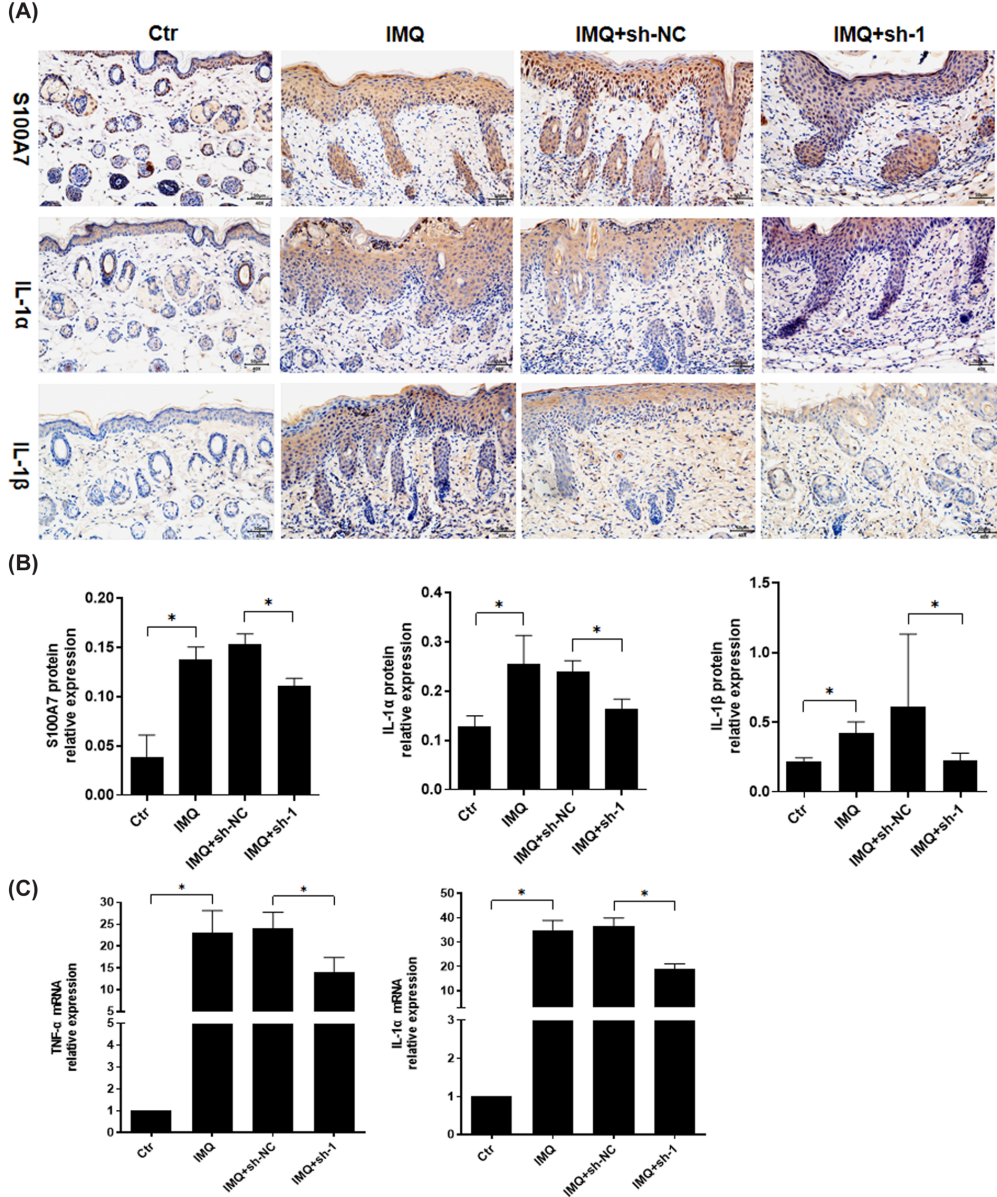
*Gpr15lg* knockdown reduced levels of inflammatory cytokines in IMQ-induced psoriatic lesions (**A**) Immunohistochemical staining and (**B**) average optical density (AOD) of IL-1α, TNF-α, IL-1β and S100A7 in mice dorsal skins. Scale bar = 200 μm. (**C**) qRT-PCR was performed to measure the expression of TNF-α mRNA and IL-1α mRNA expression in mice dorsal skin; *P<0.05.

To further investigate whether Gpr15lg can regulate immune response in psoriasis, we detected the level of IL-1α, TNF-α, IL-1β and S100A7 by qRT-PCR and IHC. As shown in Figure 3, IMQ significantly up-regulated levels of IL-1α, TNF-α, IL-1β and S100A7. However, *Gpr15lg* knockdown attenuated the up-regulation of those inflammatory cytokines. These results indicated that *Gpr15lg* knockdown could effectively ameliorate psoriasis-related inflammatory micro-environment.

### *GPR15LG* knockdown inhibited M5-induced inflammation in HaCaT cells

**Figure 4:**
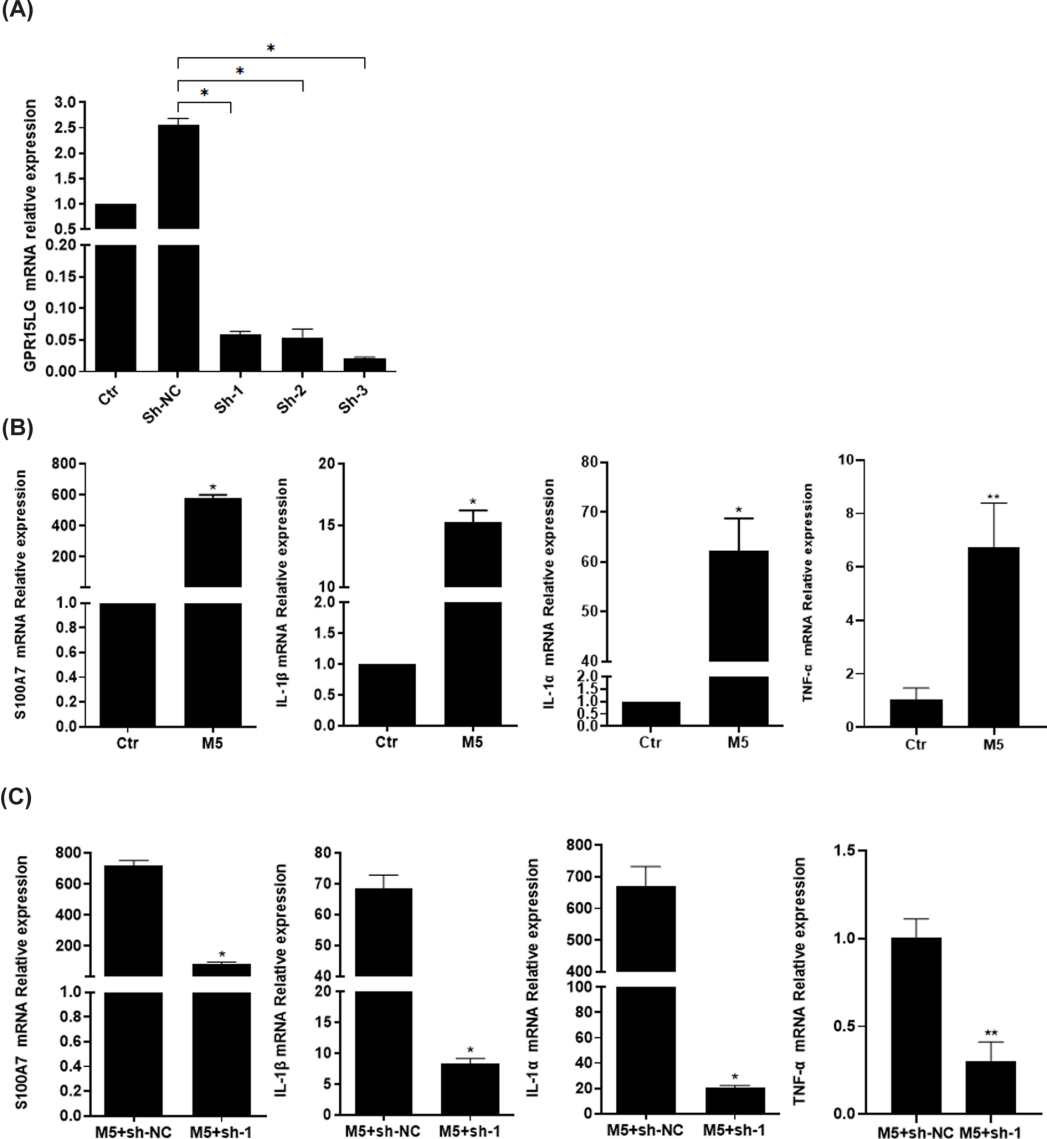
Effect of *GPR15LG* knockdown on the production of inflammatory cytokines in M5-treated HaCaT cells (**A**) *GPR15LG* mRNA expressions were determined after lentiviral particles transduction in HaCaT cells. (**B**) qRT-PCR was performed to measure the expression of IL-1α, TNF-α, IL-1β and S100A7 in control or M5-stimulated HaCaT cells. (**C**) qRT-PCR was performed to measure the expression of IL-1α, TNF-α, IL-1β and S100A7 in M5-stimulated HaCaT cells treated with sh-NC or sh-1; *P<0.05, **P<0.01.

M5 cytokines cocktail (IL-17A, IL-22, oncostatin M, IL-1α and TNF-α) was widely used to establish psoriatic cell model, and we chose this model to investigate the role of GPR15LG on the regulation of psoriasis-related cytokines *in vitro.* First, we confirmed the effective down-regulation of *GPR15LG* mRNA by shRNAs (Figure 4A). Our data showed that M5 increased the expressions of IL-1α, TNF-α, IL-1β and S100A7 (Figure 4B). However, they were all down-regulated by *GPR15LG* knockdown in M5-treated HaCaT cells (Figure 4C). These findings, which were consistent with the results *in vivo*, suggest a pivotal role for GPR15LG in keratinocyte-mediated inflammation in psoriasis.

### IL-1α and TNF-α alone promoted *GPR15LG* expression in psoriatic keratinocytes

**Figure 5:**
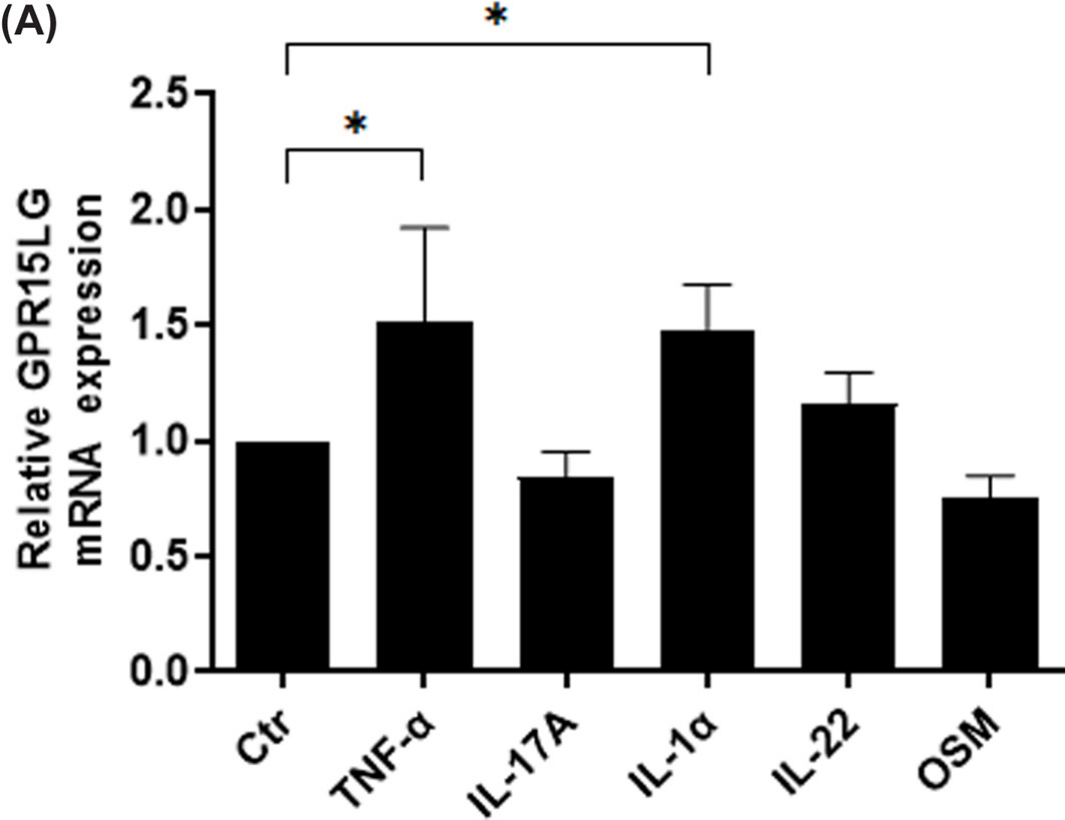
IL-1α and TNF-α alone promoted *GPR15LG* expression in psoriatic keratinocytes (**A**) *GPR15LG* mRNA expression was determined in HaCaT cell stimulated with 10 ng/ml IL-1α, TNF-α, OSM, IL-17A and IL-22 alone; *P<0.05.

We previously showed that *GPR15LG* expression was greatly elevated in M5-treated HaCaT cells, while we do not know which cytokines or cytokines could promote the expression of *GPR15LG*. In the present study, HaCaT cells were stimulated with 10 ng/ml recombinant IL-1α, TNF-α, OSM, IL-17A and IL-22 alone and we found that IL-1α and TNF-α alone could increased the expression level of *GPR15LG* mRNA in HaCaT cells (Figure 5A).

## Discussion

In the present study, we investigated the role of GPR15LG in psoriatic inflammation. We found that the knockdown of *Gpr15lg*, the mouse ortholog of *GPR15LG*, is capable of ameliorating the severity of IMQ-induced psoriatic inflammation in mice. In addition, *Gpr15lg* knockdown significantly down-regulated levels of IL-1α, TNF-α, IL-1β and S100A7 in vivo. Furthermore, *GPR15LG* knockdown inhibited M5-induced inflammation in HaCaT cells *in vitro*. The present study provided evidences that GPR15LG might participate in the progress of psoriasis via regulating keratinocyte-mediated inflammation.

GPR15LG is a multifunctional protein implicated in the pathogenesis of several diseases. GPR15LG exhibits potent wide-spectrum antimicrobial activity and it could promote cutaneous wound healing [8,9]. The role of GPR15LG in the regulation of inflammation has been previously reported. A study showed *GPR15LG* knockout mouse exhibits a decreased serum IgM level and an increased ratio of CD4+/CD8+ cells [10]. Several groups independently showed GPR15LG is a ligand for GPR15 [11–13]. It was found that *GPR15LG* is significantly elevated in psoriatic lesions [14,15]. We previously showed that GPR15LG is involved in the proliferation of psoriatic keratinocytes [14]. Recently, a study revealed a new role for GPR15LG in the inflammation and differentiation of keratinocytes [16]. Furthermore, it is an epithelial inflammation-derived pruritogen in psoriasis [17]. This line of evidence indicates that GPR15LG is critical for psoriasis development, and it may has proinflammation effect in psoriasis. However, the role it plays in psoriatic inflammation is largely unknown.

In this study, we evaluated the effects of *Gpr15lg* knockdown on IMQ-induced psoriatic inflammation in mice. The mice treated with IMQ exhibited typical psoriatic symptoms. While *Gpr15lg* knockdown significantly relieved those symptoms and improved both individual and cumulative PASI scores, and remarkably reduced the epidermal layers and inflammatory cells infiltration. Proinflammatory cytokines IL-1α, TNF-α, IL-1β and S100A7 have been reported to be up-regulated in psoriatic skins and they are involved in the psoriasis pathogenesis [6,18,19]. In our study, the strong increase of IL-1α, TNF-α, IL-1β and S100A7 was observed in psoriasis-like lesions. While *Gpr15lg* knockdown exerted an inhibitory effect on the production of these cytokines. Results indicated that *Gpr15lg* knockdown could improve IMQ-induced psoriasis-like inflammation in mice.

Evidence demonstrated that epidermal keratinocytes play crucial roles in psoriasis [20–22]. A study showed *GPR15LG* transfection increases the expression of TSLP, IL-1β, β-defensin 4, IL-6 and CXCL1 and reduces barrier gene expression in keratinocytes [16]. M5 cytokines cocktail induces keratinocytes manifesting features of psoriatic keratinocyte *in vitro* [23]. Our previous study showed that M5 cocktail greatly increased *GPR15LG* expression in HaCaT cells [14] and we chose this cell model to investigate the role of GPR15LG on psoriatic inflammation *in vitro*. We found *GPR15LG* knockdown down-regulated the expressions of IL-1α, TNF-α, IL-1β and S100A7 in M5-treated HaCaT cells, suggesting a pivotal role of GPR15LG in keratinocyte-mediated inflammation in psoriasis.

In the present study, we noted that IL-1α and TNF-α alone could induce the expression of *GPR15LG* in HaCaT cells, while more research is needed to clarified the mechanism for the induction of *GPR15LG* expression in future.

In summary, at the present study, we demonstrated that GPR15LG exhibited potent proinflammatory in psoriasis *in vivo* and *in vitro*. These results provide us with a deeper understanding of the role of GPR15LG in the pathogenesis of psoriasis at the fundamental level.

## Data Availability

The datasets used and/or analyzed during the current study are available from the corresponding author on reasonable request.

## Abbreviations

IHC: immunohistochemistry
IL: interleukin
IMQ: imiquimod
PASI: Psoriasis Area Severity Index
TNF: tumor necrosis factor
